# Shaofu Zhuyu decoction ameliorates obesity-mediated hepatic steatosis and systemic inflammation by regulating metabolic pathways

**DOI:** 10.1371/journal.pone.0178514

**Published:** 2017-06-01

**Authors:** Moonju Hong, Jeeyoun Jung, Hee-Sook Park, So Min Lee, Nam-Joo Jeong, Soon-Hee Kim, Kyoung-Won Lee, Ju-A Lee, Myung-Sunny Kim

**Affiliations:** 1 Division of Metabolism and Nutrition, Korea Food Research Institute, Gyeonggi-do, Republic of Korea; 2 Department of Food Biotechnology, Korea University of Science & Technology, Gyeonggi- do, Republic of Korea; 3 KM Fundamental Research Division, Korea Institute of Oriental Medicine, Yuseong-Gu, Daejeon, Republic of Korea; Bambino Gesù Children's Hospital, ITALY

## Abstract

Shaofu Zhuyu decoction (SFZYD, also known as Sobokchugeo-tang), a classical prescription drug in traditional East Asian medicine, has been used to treat blood stasis syndrome (BSS). Hepatic steatosis is the result of excess caloric intake, and its pathogenesis involves internal retention of phlegm and dampness, blood stasis, and liver Qi stagnation. To evaluate the effects of treatment with SFZYD on obesity-induced inflammation and hepatic steatosis, we fed male C57BL/6N mice a high fat diet (HFD) for 8 weeks and then treated them with SFZYD by oral gavage for an additional 4 weeks. The results of histological and biochemical examinations indicated that SFZYD treatment ameliorates systemic inflammation and hepatic steatosis. A partial least squares-discriminant analysis (PLS-DA) scores plot of serum metabolites showed that HFD mice began to produce metabolites similar to those of normal chow (NC) mice after SFZYD administration. We noted significant alterations in the levels of twenty-seven metabolites, alterations indicating that SFZYD regulates the TCA cycle, the pentose phosphate pathway and aromatic amino acid metabolism. Increases in the levels of TCA cycle intermediate metabolites, such as 2-oxoglutaric acid, isocitric acid, and malic acid, in the serum of obese mice were significantly reversed after SFZYD treatment. In addition to inducing changes in the above metabolites, treatment with SFZYD also recovered the expression of genes related to hepatic mitochondrial dysfunction, including *Ucp2*, *Cpt1α*, and *Ppargc1α*, as well as the expression of genes involved in lipid metabolism and inflammation, without affecting glucose uptake or insulin signaling. Taken together, these findings suggest that treatment with SFZYD ameliorated obesity-induced systemic inflammation and hepatic steatosis by regulating inflammatory cytokine and adipokine levels in the circulation and various tissues. Moreover, treatment with SFZYD also reversed alterations in the levels of metabolites of the TCA cycle, the pentose phosphate pathway and aromatic amino acid metabolism.

## Introduction

Traditional East Asian medicines have long been used to treat various diseases. These treatment modalities are designed to heal a person through a holistic approach and to balance internal vital energy. Herbal remedies consist of various natural herbs, minerals, and animal products, and each of these elements contains a variety of chemical compounds [1], each of which can target different causes of disease progression. Shaofu Zhuyu decoction (SFZYD, Sobokchugeo-tang in Korean) is used as a blood stasis syndrome (BSS) remedy in traditional Korean medicine (TKM) and traditional Chinese medicine (TCM). SFZYD was originally described in “Correction of Errors in Medical Classics”, which was compiled by Qing-ren Wang during the Qing dynasty of China (A.D. 1830), and consists of ten crude herbs [2, 3]. SFZYD is widely used in clinical practice to treat BSS in gynecologic diseases, such as primary dysmenorrhea of cold coagulation, blood stasis and menoxenia [2, 3]. In traditional East Asian medicine, BSS has reportedly been linked to disturbances in blood circulation and microcirculation, changes in blood physical and chemical properties, abnormal tissue hyperplasia, endothelial cell dysfunction, metabolic disorders, inflammation, and immune dysfunction [4]. We recently demonstrated that Tongqiaohuoxue (THD) decoction, which is used to treat blood stasis and hypercoagulation in traditional medicine, ameliorates obesity-induced inflammation and prothrombotic states [5], and emerging evidence indicates that BSS remedies have anti-inflammatory properties and primary effects in blood stasis.

Excess intake of high-fat foods results in hepatic steatosis, which, according to East Asian medicine, is induced by blood stasis, internal retention of phlegm and dampness, liver Qi stagnation, and deficient spleen or kidney function [6]. Hepatic steatosis has several causes. The two main causes of the disease are hepatocellular triglyceride (TG) accumulation resulting from lipid uptake imbalances and chronic inflammation. Hepatic steatosis is promoted by hepatic lipid dysregulation, oxidative stress, mitochondrial dysfunction and chronic systemic inflammation otherwise induced by pro-inflammatory cytokines, such as monocyte chemoattractant protein-1 (MCP-1), tumor necrosis factor-α (TNF-α), and interleukin-6 (IL-6), secreted by the liver and adipocytes [7, 8, 9, 10, 11]. Inflammation is associated with important pathogenic mechanisms that are involved in the development of obesity-associated hepatic steatosis and blood stasis. In this study, we demonstrated the efficacy of SFZYD in an obesity-induced hepatic steatosis model and elucidated the mechanism underlying its effects.

Metabolomics has recently attracted interest in biomarker discovery and has been shown to be useful for assessing the holistic therapeutic effects of many multi-component pharmaceuticals and TKM or TCM remedies whose most prominent characteristics enable their use in holistic approaches, as well for examining the function and dysfunction of living organs [12]. Because multiple compounds interact with multiple targets with interdependent activities *in vivo*, metabolomics coupled with multiple statistical tools focusing on comprehensive analyses of small molecules and biological pathways is a valuable approach for understanding the effects of traditional herbal medicines, as it not only enables elucidation of the relationships between phenotype and metabolism but also identifies the key metabolites associated with a particular phenotype. In this study, we used metabolomics analysis to investigate the beneficial effects of SFZYD in systemic inflammation and hepatic steatosis in diet-induced obese (DIO) mice. We analyzed the metabolic phenotypes of these mice and used a capillary electrophoresis time of flight mass spectrometry (CE-TOF/MS)-based metabolic approach to elucidate the physiological function of SFZYD in obesity.

## Materials and methods

### SFZYD preparation

We prepared SFZYD decoction by extracting a mixture comprising the following 10 dried medicinal herbs: 4 g of *Foeniculum vulgare* Mill., 0.8 g of *Zingiber officinale* Roscoe, 4 g of *Corydalis ternata* Nakai, 4 g of *Commiphora molmol* Engler, 12 g of *Angelica gigas* N., 4 g of *Cnidium officinale* Makino, 4 g of *Cinnamomum loureirii* Nees, 8 g of *Paeonia obovata* Maxim., 12 g of *Typha angustifolia* L., and 8 g of *Trogopterus xanthipes*. All the herbal components were purchased from Omniherb (Daegu, Korea) and were inspected by Jun-Kyung Lee of the Hyemin Dispensary of Oriental Medicine, Korea. The decoction was extracted from a mixture of chopped crude herbs by reflux extraction (COSMOS-660, Kyungseo Machine CO. Incheon, Korea) in distilled water for 3 hrs at 100°C. The extract was freeze-dried to make a powder (Extraction yield of 13.04%) and then stored at -70°C.

### HPLC analysis

**Fig 1:**
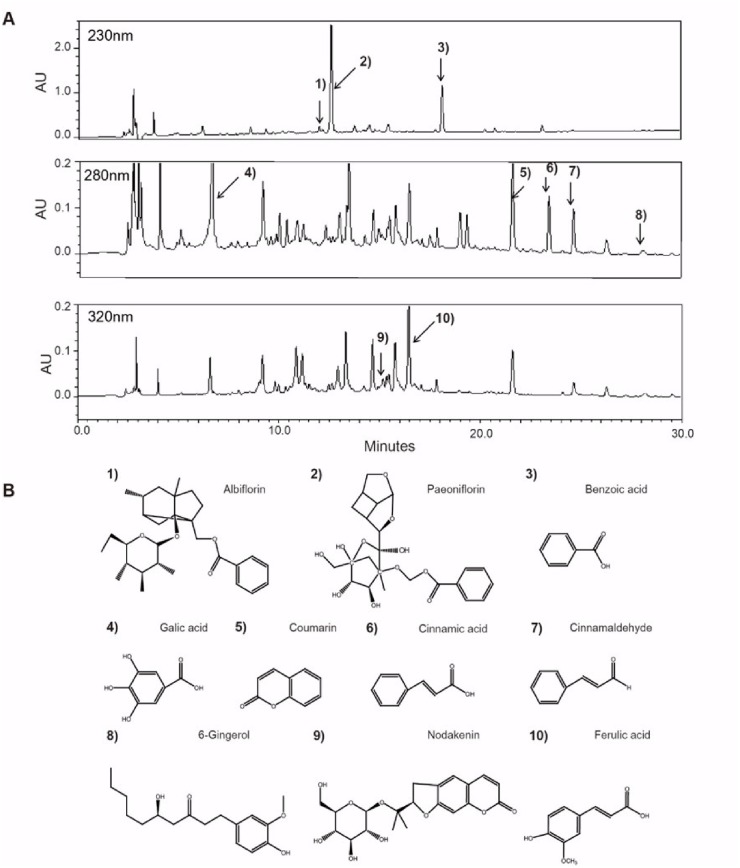
Chemical profile of the SFZYD extract. (A) HPLC chromatography of SFZYD. (B) Chemical structures of the ten compounds constituting SFZYD. Key for chromatography: 1. alboflorin, 2. peoniflorin, 3. benzoic acid, 4. gallic acid, 5. coumarin, 6. cinnamic acid, 7. cinnamaldehyde, 8. 6-gingerol, 9. nodakenin, and 10. ferulic acid.

The chemical profile of SFZYD was determined by high-performance lipid chromatography (HPLC). Standard chemicals (alboflorin, peroniflorin, benzoic acid, gallic acid, coumarin, cinnamic acid, cinnamaldehyde, 6-gingerol, nodakenin, and ferulic acid) were purchased from NPC BioTechnology Inc. (Daejeon, Korea), and HPLC-grade solvents were obtained from J.T. Baker (Phillipsburg, NJ, USA). HPLC was conducted using a Waters Alliance 2695 system coupled with a 2998 photodiode array detector, and data processing was performed with Empower software (Waters Co., Milford, MA, USA). Chromatographic separation was accomplished using a Luna 5 m C18 (2) 100 A (4.6 x 250 mm, 5-μm particle size, No. 00G-4252-E0, Phenomenex Co., Torrance, CA, USA) column. The elution was monitored at the following wavelengths: 230 nm for the albiflorin, paeoniflorin, and benzoic acid components; 280 nm for the gallic acid, coumarin, chinnamic acid, and cinnamaldehyde components; and 320 nm for 6-gingerol, nodakenin, and ferulic acid components. The mobile phases consisted of water with 0.1% (v/v) trifluoroacetic acid (solvent A) and acetonitrile (solvent B) at a flow rate of 1.0 ml/min. The following gradient (A to B) was used: 0–40 min at 5–60% B. The chemical fingerprints of SFZYD were analyzed by HPLC and compared to those of the above standard chemical compounds, and HPLC was performed to identify the bioactive components in the SFZYD extract. Ten major bioactive components were identified, according to the relative retention time of each standard (Fig 1).

### Animals and diets

This study was performed in strict accordance with the appropriate guidelines and was approved by the Institutional Animal Care and Use Committee of the Korea Food Research Institute (#KFRI-M-14006). Five-week-old male C57BL/6NCrSlc mice purchased from Japan SLC, Inc. (Hamamatsu, Japan) were housed at KFRI at a controlled temperature (24.0 ± 1°C with a relative humidity of 50 ± 5%) under 12-hr light/dark cycles and were allowed access to food and water *ad libitum*. After adapting to the above environment for 1 week, the mice were divided into two groups and fed a 10% kcal normal chow diet (NC; D12450B, Research Diets, New Brunswick, NJ, USA) or a 60% kcal high-fat diet (HFD; D12492) for 8 weeks. The HFD group was then separated into the following two groups: an HFD group that was administered SFZYD orally at a dose of 100 mg/kg body weight once a day for 4 weeks, and an HFD group that was administered water as a vehicle control. Body weight and food intake were measured every week. Blood was collected from the retroorbital sinus just before the mice were sacrificed, after which serum samples were prepared. Harvested liver and epididymal adipose tissue (AT) samples were then weighed, immediately frozen in liquid nitrogen and stored at -80°C until analysis.

### Histological examination

Liver tissue specimens were fixed in 4% formalin, embedded in paraffin and then cut into 4-μm sections. The sections were subsequently stained with hematoxylin and eosin (H&E), after which images were captured using the indicated microscope (ECLIPSE 80i, Nikon, Japan).

### Biochemical analysis of serum and liver tissues

Total white blood cell (WBC) counts were measured using an automated cell counter (Hemavet 950 FS, Drew Scientific Inc., Waterbury, CT, USA). Serum was separated from blood by centrifugation at 12,000 rpm for 2 min, and serum MCP-1 and adipokine levels were determined using enzyme-linked immunosorbent assay (ELISA) kits, according to the manufacturer’s instructions. The adiponectin and leptin kits were obtained from ALPCO Diagnostics (Salem, NH, USA), and the MCP-1 kit was purchased from Enzo Diagnostics (Farmingdale, NY, USA).

### Analysis of serum metabolites

Serum metabolite analysis was performed by Human Metabolome Technologies, Inc. (HMT; Tsuruoka, Japan). Briefly, serum was extracted, filtered, centrifuged, and then subjected to metabolomics analysis using an Agilent capillary electrophoresis time of flight mass spectrometry (CE-TOF/MS) system (Agilent Technologies Inc., Santa Clara, CA, USA). All the samples were analyzed in cation and anion mode. Processed peak data detected via CE-TOF/MS analysis were extracted using automatic integration software (MasterHands, ver. 2.13.0.8.h, Keio University-developed software) to obtain the following information: m/z values, migration times (MTs) and areas [13]. Each peak was aligned according to migration time on CE, and m/z values were determined by TOF-MS. Putative metabolites were identified using the appropriate database based on the m/z and MT data. The tolerances were ± 0.5 min in MT and ± 10 ppm in m/z, and the metabolite concentrations were calculated by normalizing the peak areas to the peak area of the internal standard.

### Multivariate analysis for serum metabolite profiling

Quantitative data pertaining to specific serum metabolites were imported to SIMCA-P version 14.0 (Umetrics, Umea, Sweden). Prior to multivariate data analysis, the variables were centered and scaled using unit-variance scaling, a process in which each variable was divided by the standard deviation of the column values [14]. First, principal component analysis (PCA) was used as an unsupervised pattern recognition method to determine whether the serum metabolic profiles of the different groups analyzed herein featured inherent similarities, and a loading plot was generated to identify the metabolites responsible for the differences in the PCA score plots. Then, partial least-squares discriminant analysis (PLS-DA) was conducted to maximize the covariance between the measured data (serum metabolite profiles) and the response variables (predicted classifications), and the reliability of the PLS-DA model was validated by a CV-ANOVA test and a 100-fold repeated permutation test using SIMCA-P software [15]. The quality of the PCA and PLS-DA models was described by *R*^*2*^ and *Q*^*2*^ values. *R*^*2*^ was defined as the proportion of variance in the data explained by the models and indicated goodness of fit, and *Q*^*2*^ was defined as the proportion of variance in the predicted data identified by the model and indicated predictability.

### Metabolite pathway analysis

Metabolite pathway analysis was performed in MetaboAnalyst Pathway Analysis (http://www.metaboanalyst.ca/Metabo-Analyst/), based on database sources, including the Kyoto Encyclopedia of Genes and Genomes database (KEGG; http://www.genome.jp/kegg/), to identify, analyze, and visualize the metabolic pathways affected by the above treatments. The *p* value was calculated by enrichment analysis, and the pathway impact (PI) value was calculated by pathway topology analysis. Furthermore, a heatmap, which is commonly used for unsupervised clustering, was generated in MetaboAnalyst 3.0 (http://www.metaboanalyst.ca) using data pertaining to the metabolites whose levels differed significantly among the 3 groups.

### Correlation network

A correlation network was generated using the significant correlations (*p* value < 0.05) between serum metabolite levels and blood parameter levels or between serum metabolite levels and liver and AT gene mRNA expression levels, which were calculated using PASW, version 18.0 for Windows (SPSS Inc. Chicago, IL, USA). We utilized an edge-weighted spring-embedded layout algorithm in Cytoscape 3.2.1. The edges were weighted according to the association—log (*p*) values.

### RNA extraction and quantitative real-time PCR

Total RNA was isolated from liver and epididymal AT using RNeasy Lipid Tissue Mini Kit (Qiagen, Valencia, CA, USA) protocols and reagents, and first-strand cDNA was synthesized from 1 μg of total RNA using an iScriptTM cDNA Synthesis Kit (Bio-rad, Hercules, CA, USA). Quantitative real-time PCR (qRT-PCR) was performed on a LightCycler^®^ 480 Real-Time PCR System (Roche Diagnostics, Mannheim, Germany) using TaqMan Universal Master Mix II and TaqMan probes (Life Technologies, Foster City, CA, USA). The PCR protocol comprised the following steps: 50°C for 2 min and 95°C for 10 min, followed by 40 cycles at 95°C for 15 s and 60°C for 1 min. The following TaqMan probes were used for these experiments: *Ccl2* (MCP-1), *Tnfα*, *Il1β*, *Lpl*, *Fabp4* (aP2), *PPARγ*, *Ucp2*, *Cpt1α*, *Ppargc1α* (PGC-1α), *Il6*, *Serpine1* (PAI-1), *AdipoQ* (Adiponectin), *Leptin*, *Slc2a4* (GLUT4), and *Irs1* (Life Technologies, Foster City, CA, USA). Each experiment was performed in duplicate, and the relative expression levels of the genes in question were normalized to those of *18S* or *Tbp* mRNA using the ΔCt method.

### Statistical analysis

All results are expressed as the mean ± SEM and were analyzed using unpaired t-tests and one-way analysis of variance (ANOVA), followed by Tukey’s post hoc comparison. Differences were considered statistically significant at *p* < 0.05 and were stratified further according to whether their corresponding *p* values were as follows: *p* < 0.01 and *p* < 0.001. All statistical analyses were analyzed using GraphPad Prism 5 (GraphPad Prism Software, Inc. San Diego, CA, USA).

## Results

### SFZYD treatment ameliorates HFD-induced hepatic steatosis and systemic inflammation

**Fig 2:**
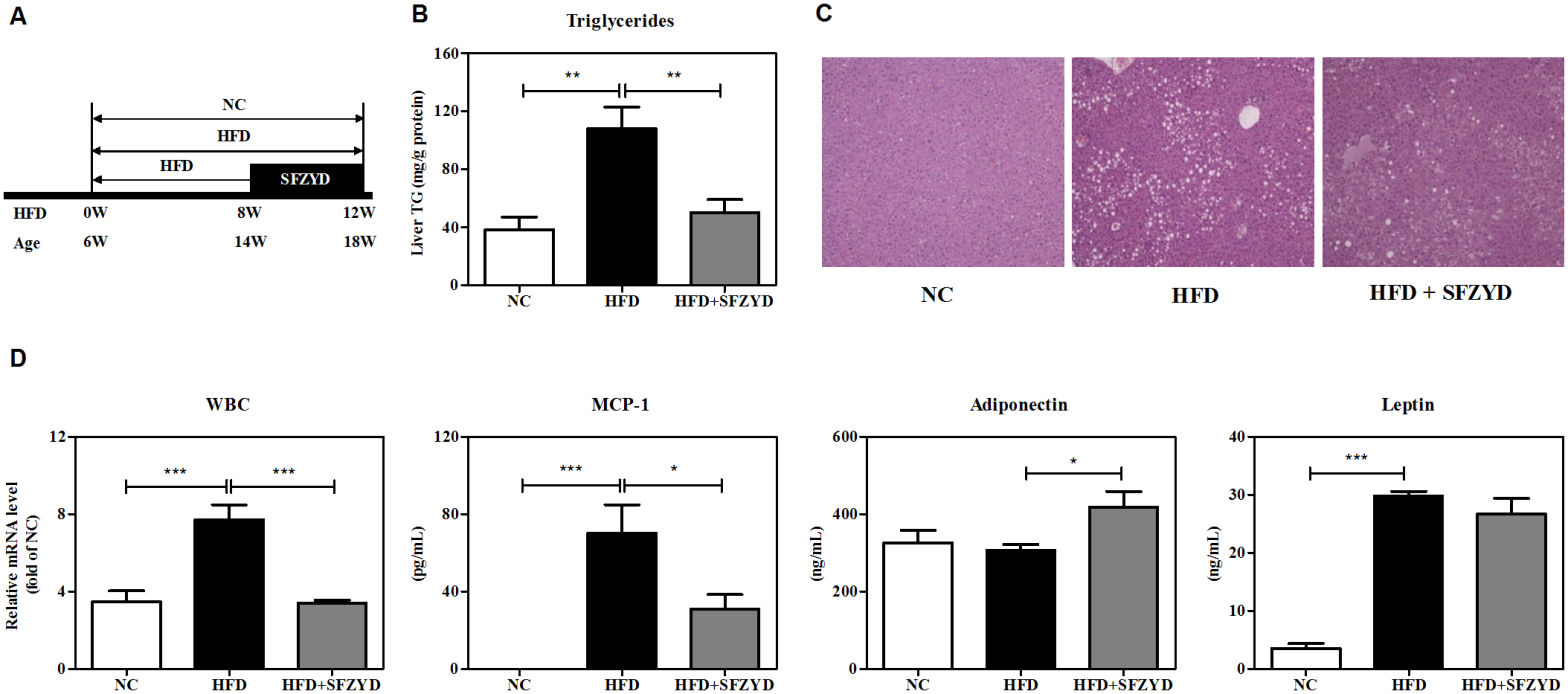
SFZYD treatment ameliorates HFD-induced hepatic steatosis and systemic inflammation. (A) Experimental scheme of SFZYD treatment in HFD-induced obese mice (B) Hepatic TG levels (C) Representative liver tissues stained with H&E (D) Total WBC levels in blood and MCP-1, adiponectin, and leptin levels in serum. Data are expressed as the mean ± SEM of 6–8 mice per group. *, *p* < 0.05; **, *p* < 0.01; ***, *p* < 0.001.

To demonstrate the effects of SFZYD treatment in hepatic steatosis and inflammation, we fed C57BL/6N mice an NC diet or an HFD (60% kcal fat) for 8 weeks. The HFD group was divided into two groups that were fed an HFD or an HFD+SFZYD (100 mg/kg/day) orally for an additional 4 weeks (Fig 2A). The hepatic TG concentration in the liver of HFD-fed obese mice was significantly increased compared to that of NC mice. Treatment with SFZYD treatment significantly reduced the TG concentration to a normal level (Fig 2B). Serum TG levels were also significantly reduced by SFZYD treatment (Table 1). Consistent with this result, the results of the histological analysis of the liver showed that lipid droplet accumulation in hepatocytes was decreased by SFZYD treatment in the indicated group compared to the NC group (Fig 2C). We also examined the levels of various inflammation-related markers in the circulation. The total WBC in blood was decreased by SFZYD treatment compared to HFD treatment. SFZYD treatment also down-regulated MCP-1 expression levels in the serum of the corresponding group of mice compared to the HFD-fed group of mice (Fig 2D). The decreases in serum adiponectin levels that occurred in HFD-fed mice were reversed by SFZYD treatment (Fig 2D). The post-SFZYD treatment level of unconverted leptin in the serum of HFD-fed mice was lower than the corresponding level in untreated HFD-fed mice, and the difference in the level was indicative of differences in stationary body fat, epididymal fat, and subcutaneous fat weight between the two groups (Fig 2D, Table 1). No significant differences in liver or spleen weight were observed between the HFD group and the SFZYD-treated HFD group (Table 1).

**Table 1:** Phenotypes in HFD-induced obese mice treated with SFZYD.

	NC	HFD	HFD+SFZYD
**N**	8	7	7
**Fasting body weight (g)**	27.54 ± 10.48^a^	39.88 ± 0.35^b^	40.40 ± 0.41^b^
**Serum triglycerides (mg/dL)**	133.18 ± 9.16 ^a^	122.05 ± 5.50 ^ab^	106.74 ± 2.59 ^b^
**Organ weights**			
**Liver (g)**	0.99 ± 0.03^a^	1.26 ± 0.09^b^	1.25 ± 0.05^ab^
**Epididymal fat (g)**	0.63 ± 0.09^a^	1.83 ± 0.15^b^	1.88 ± 0.12^b^
**Subcutaneous fat (g)**	0.42 ± 0.06^a^	2.14 ± 0.11^b^	1.96 ± 0.13^b^
**Spleen (g)**	0.07 ± 0.00^a^	0.11 ± 0.02^a^	0.08 ± 0.00^a^

Data are expressed as the mean ± SEM. The significance of the differences among the three groups was calculated using one-way ANOVA followed by Tukey’s test. Values with no common superscript letter were significantly different from the corresponding values in the other two groups at *p* < 0.05.

### SFZYD alters the metabolic patterns of the HFD-induced obesity model

**Fig 3:**
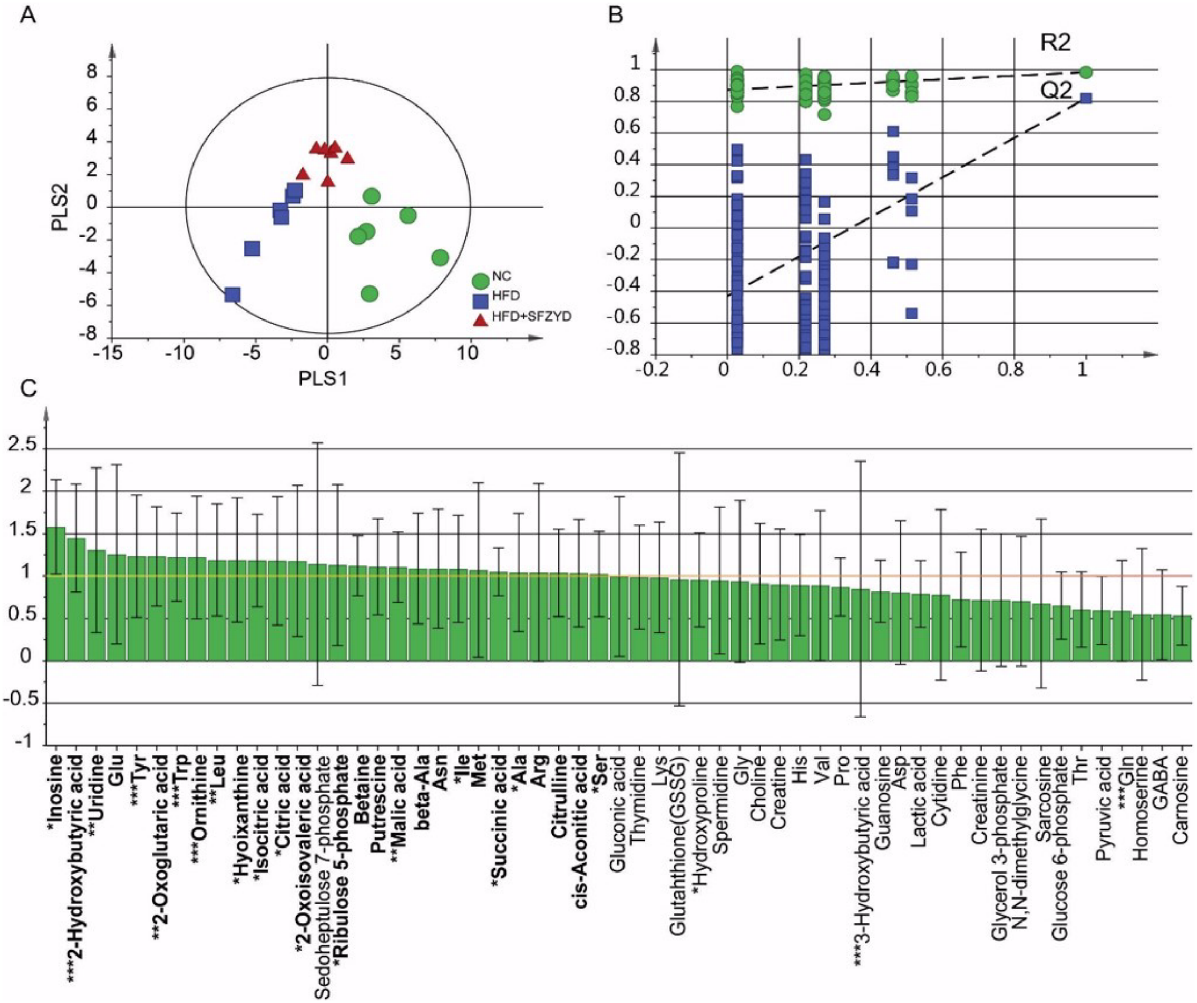
PLS-DA and loading plots for metabolic profiling of serum. (A) PLS-DA score plots. (B) Permutation test (repeated 100 times). (C) VIP plot with jack-knifed confidence intervals derived from the concentrations of the indicated serum metabolites. In the VIP plot, the bold letters indicate the metabolites that significantly contributed to the PLS-DA model (VIP > 1), and the asterisk (*) indicates the metabolites with a *p* value lower than 0.05 in the ANOVA test.

We performed metabolomic analyses of 26 samples of murine serum using CE-TOF/MS. One hundred sixty-nine putative metabolites (103 metabolites in cation mode and 66 metabolites in anion mode) were identified via the HMT metabolite database, and the expression of 57 metabolites was quantified using standard HMT metabolites. To assess the differences in the metabolic patterns of the three groups, we performed PLS-DA to minimize the possible effects of intergroup variability on the results of the analysis and improve the separation among the mice in the NC, HFD, and SFZYD-treated HFD groups (Fig 3A). The score plots from the PLS-DA model showed that there were clear differences in the metabolic patterns of the three groups (R^2^Y = 0.973 and Q^2^ = 0.757) and that the *p* value pertaining to these differences was lower than 0.05 in the CV-ANOVA test (*p* = 0.015). Similarly, the PCA score plots derived from data pertaining to the serum concentrations of the indicated metabolites in NC, HFD, and SFZYD-treated HFD mice showed that the metabolites produced by HFD mice were similar those produced by NC mice after SFZYD administration (R2X = 0.594 and Q2 = 0.158; S1 Fig). In addition, we validated the reliability of the model using a 100-fold repeated permutation test (Fig 3B). Among the 57 metabolites whose expression was quantified, 27 contributed to the PLS-DA model (variable importance of projection (VIP) > 1 and jack-knifing of VIP; Fig 3C). In particular, inosine, 2-hydroxybutyric acid, uridine, Tyr, 2-oxoglutaric acid, Trp, ornithine, Leu, hypoxanthine, isocitric acid, citric acid, 2-oxoisovaleric acid, ribulose 5-phosphate, malic acid, Ile, succinic acid, Ala, Ser, hydroxyproline, 3-hydroxybutyric acid and Gln exhibited significant differences in their expression levels among the three groups (*p* < 0.05 in ANOVA or non-parametric ANOVA tests; Fig 3C and Table 2).

**Table 2:** Serum metabolites exhibiting significant differences in their expression among the three groups, as determined by comparative analysis.

		Comparative analysis					
		HFD vs NC			HFD+SFZYD vs HFD		
KEGG pathway information	Metabolite†	Ratio ^¶^	*p*-value		Ratio ^¶^	*p*-value	
**Central carbohydrate metabolism (TCA cycle)**	2-Oxoglutaric acid**,§	1.6	0.07		0.6	0.009	&&
	Citric acid*	1.2	0.06		0.8	0.012	&
	Isocitric acid*,§	1.1	0.22		0.8	0.009	&&
	Malic acid**	1.6	0.01	&&	0.7	0.034	&
	Succinic acid*,§	1.7	0.03	&	0.9	0.116	
**Central carbohydrate metabolism (Pentose phosphate pathway)**	Ribulose 5-phosphate*	1.5	0.04	&	0.7	0.072	
**Nucleotide metabolism (Purine, pyrimidine metabolism)**	Inosine***	1.8	0.02	&	0.2	<0.001	&&&
	Uridine**	1.2	0.04	&&	1	0.977	
	Hypoxanthine*	1.3	0.08		0.3	0.018	&
**Carbohydrate metabolism (Propanoate metabolism)**	2-Hydroxybutyric acid***,	0.4	<0.001	&&&	1.2	0.678	
**Lipid metabolism (Synthesis and degradation of ketone bodies)**	3-Hydroxybutyric acid***,	0.4	<0.001	&&&	1.5	0.037	&
**Amino acid metabolism (Branched-chain amino acid metabolism)**	Leu**	0.8	0	&&	1.1	0.41	
	Ile*	0.8	0.01	&	1.1	0.672	
	2-Oxoisovaleric acid*	1.1	0.62		0.9	0.034	&
**Amino acid metabolism (Alanine metabolism)**	Ala*	1.3	0.02	&	0.9	0.705	
**Amino acid metabolism (Arginine biosynthesis, arginine and proline metabolism)**	Gln***	1	1		1.1	<0.001	&&&
	Ornithine***	1.7	<0.001	&&	0.9	0.447	
	Hydroxyproline*	0.7	0.03	&	1.2	0.54	
**Amino acid metabolism (Glycine, serine, and threonine metabolism)**	Ser*	1.2	0.02	&	0.9	0.257	
**Amino acid metabolism (Aromatic amino acid metabolism)**	Trp***	1.7	<0.001	&&&	0.9	0.236	
	Tyr***,§	1.6	0.01	&	0.7	0.012	&

^†^, The asterisks after the metabolites indicate the results of one-way ANOVA tests,

*, *p* < 0.05,

**, *p* < 0.01,

***, *p* < 0.001;

^§^, Non-parametric test;

^¶^, In the ratio calculations, the latter is the denominator;

^&^, *p* < 0.05,

^&&^, *p* < 0.01,

^&&&^, *p* < 0.001

### SFZYD treatment alters energy patterns and purine, pyrimidine and aromatic amino acid metabolism in obese mice

**Fig 4:**
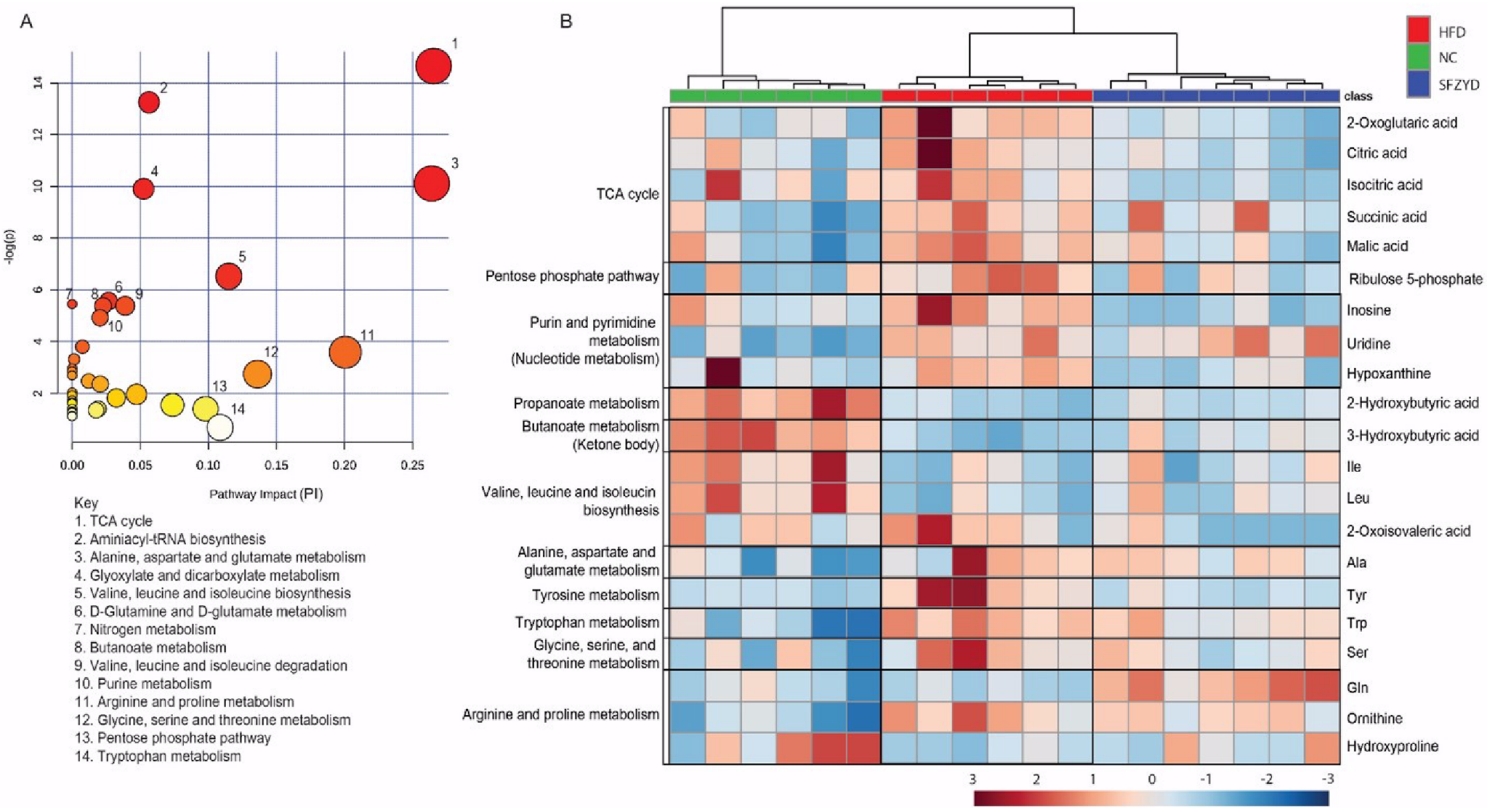
SFZYD treatment alters metabolic patterns. (A) Metabolic pathway analysis. (B) Heatmap visualization of the metabolites whose levels were significantly altered in ANOVA. The keys in the metabolic pathway analysis indicated the metabolic pathway with—log (*p*) > 4.5 or PI > 0.1. The annotated pathway for each metabolite in the heatmap was the pathway that had the highest PI, as determined by pathway topology analysis.

To gain insights into the metabolic patterns of SFZYD-treated HFD mice, we performed metabolic pathway analysis of the metabolites whose expression levels were significantly altered by SFZYD treatment using the ‘‘pathway analysis” module within MetaboAnalyst software. Pathway analysis showed that the metabolites in question were involved in the following processes: TCA cycle progression; aminoacyl-tRNA biosynthesis; alanine, aspartate and glutamate metabolism; glyoxylate and dicarboxylate metabolism; valine, leucine and isoleucine biosynthesis; D-glutamine, D-glutamate, nitrogen, and butanoate metabolism; valine, leucine and isoleucine degradation; purine, arginine and proline metabolism; glycine, serine and threonine metabolism; pentose phosphate pathway activity; and tryptophan metabolism [–log (*p*) > 4.5 or pathway impact (PI) > 0.1] (Fig 4A). Furthermore, heatmap visualization, which is commonly used in unsupervised clustering, was implemented to determine the distributions of the metabolites whose levels were significantly altered by SFZYD treatment and were thus significantly different among the 3 groups (Fig 4B). The levels of the metabolites involved in the TCA cycle, the pentose phosphate pathway, and purine metabolism in HFD mice were increased compared to those in NC mice, whereas the levels of the metabolites involved in the above processes in SFZYD-treated HFD mice were decreased compared to those in HFD mice (Table 2). In addition, malic acid, inosine, and Tyr levels and 3-hydroxybutyric acid levels in HFD mice were significantly higher and lower, respectively, than those in NC mice. These changes were significantly reversed by SFZYD administration (Table 2).

### SFZYD treatment regulates the expression of genes involved in inflammation, lipid metabolism and mitochondrial dysfunction

**Fig 5:**
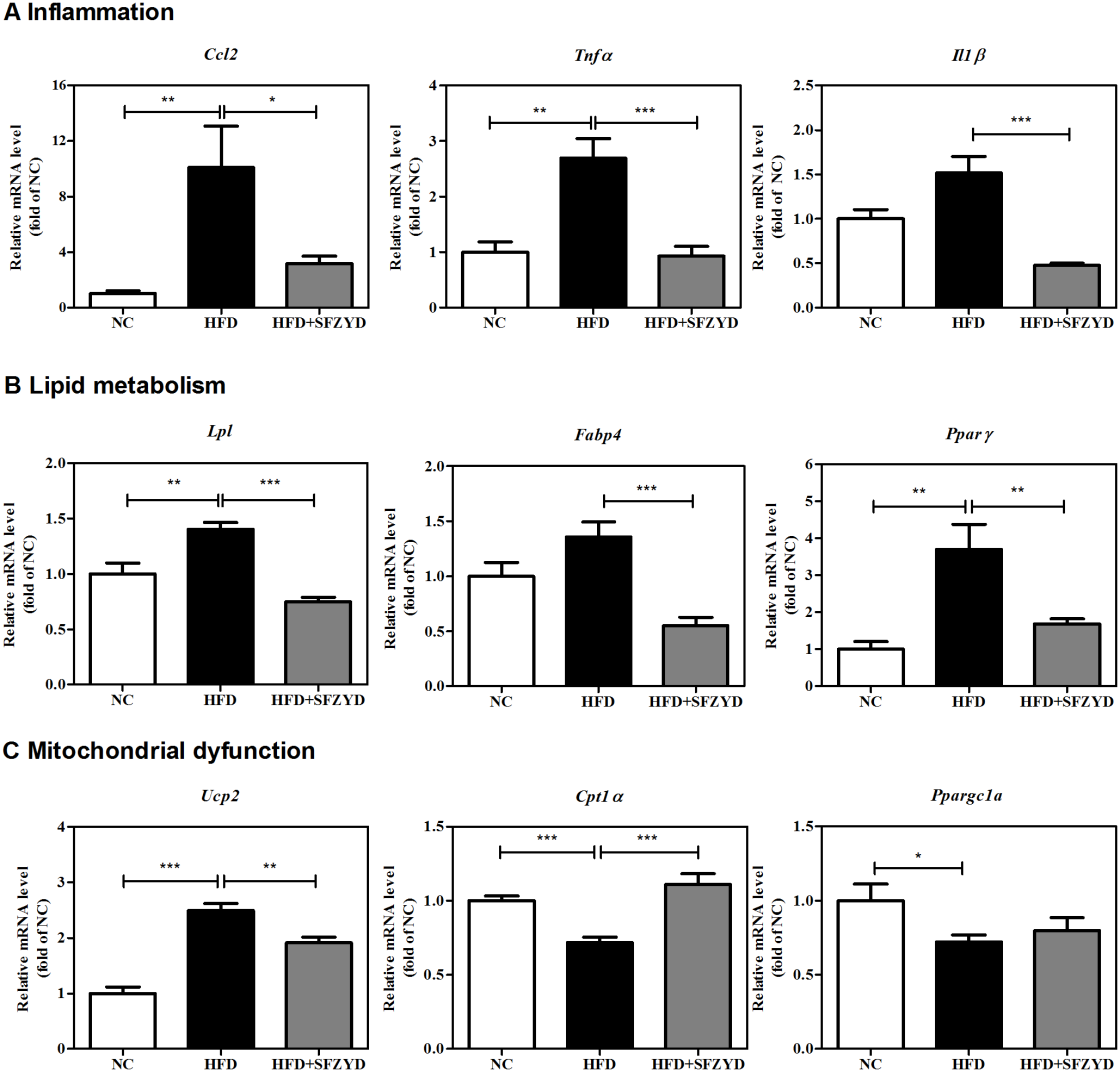
SFZYD treatment regulates the expression of the hepatic genes involved in inflammation, lipid metabolism, and mitochondrial dysfunction. mRNA levels in liver tissue were evaluated by real-time RT-PCR analysis. (A) The relative expression levels of genes related to inflammation (*Ccl2*, *Tnfα*, and *Il1β*). (B) The relative expression levels of genes related to lipid metabolism (*Lpl*, *Fabp4*, and *Pparγ*). (C) The relative expression levels of genes related to mitochondrial dysfunction (*Ucp2*, *Cpt1α* and *Ppargc1α*). Data are expressed as the mean ± SEM for 6–8 mice per group. *, *p* < 0.05; **, *p* < 0.01; ***, *p* < 0.001.

The hepatic mRNA expression levels of *Ccl2*, *Tnfα*, and *Il1β*, which are related to inflammation, were significantly decreased and increased in SFZYD-treated HFD mice and HFD mice, respectively, compared to NC mice (Fig 5A), and the mRNA expression levels of *Lpl*, *Fabp4*, and *Pparγ*, which are related to lipid metabolism, were significantly reduced after SFZYD administration in the corresponding group compared to the HFD control group (Fig 5B). In addition, the changes in the expression levels of *Ucp2*, *Cpt1α* and *Ppargc1α*, which are related to mitochondrial dysfunction in fatty liver, were attenuated after SFZYD administration (Fig 5C).

**Fig 6:**
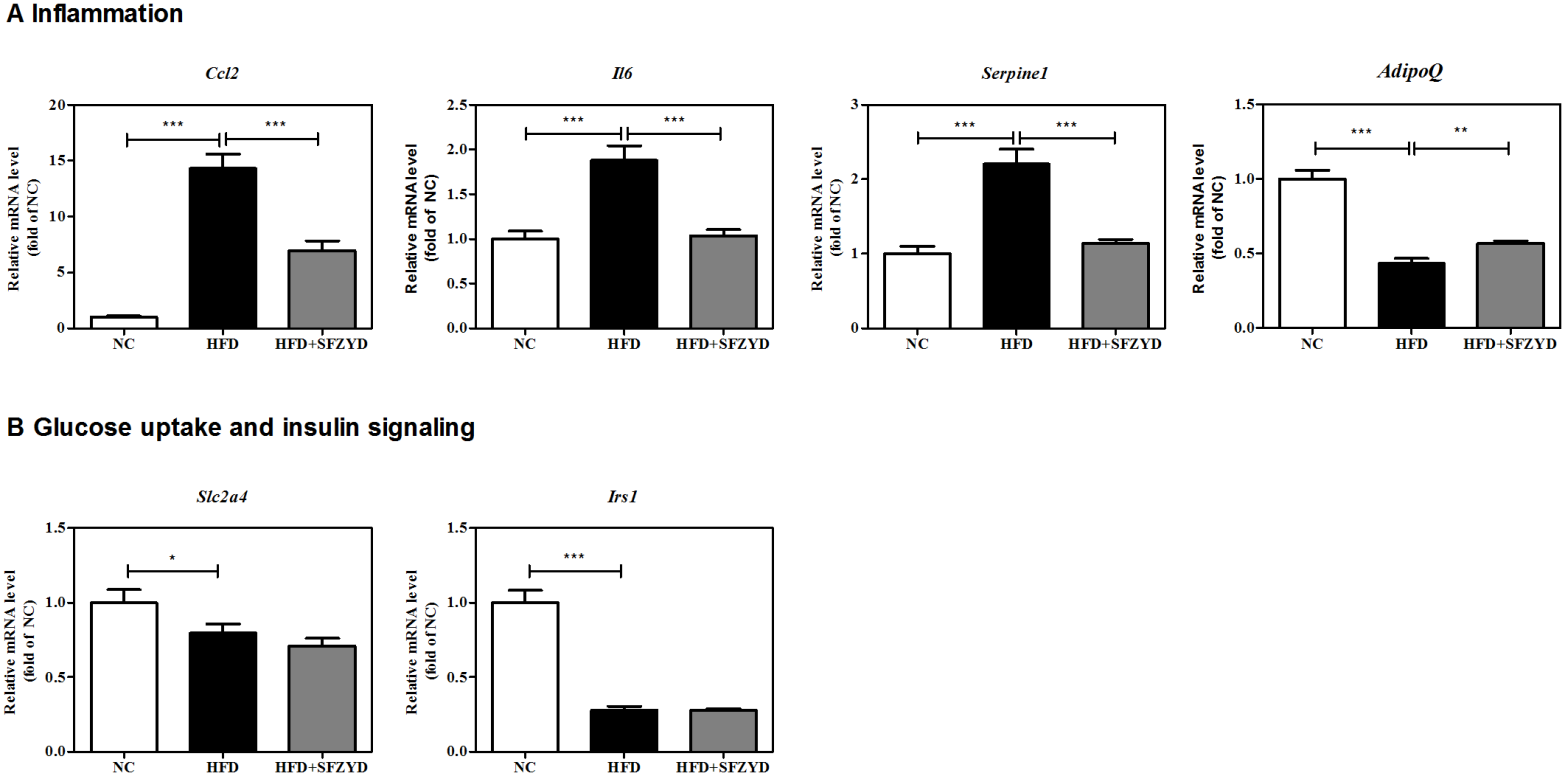
SFZYD treatment regulates inflammatory and adipokine expression without affecting insulin signaling in adipose tissue. The mRNA expression levels of several inflammatory markers and adipokines in AT were evaluated by real-time RT-PCR analysis. (A) The relative expression levels of genes related to inflammation (*Ccl2*, *Il6*, *Serpine1*, and *AdipoQ*) (B) The relative expression levels of genes related to glucose uptake and insulin signaling (*Slc2a4* and *Irs1*). Data are expressed as the mean ± SEM for 6–8 mice per group. *, *p* < 0.05; **, *p* < 0.01; ***, *p* < 0.001.

Inflammatory gene down-regulation also occurred in AT. The expression levels of various inflammatory markers, namely, *Ccl2*, *Il6* and *Serpine1*, were significantly decreased and increased in SFZYD-treated HFD mice and HFD mice, respectively, compared to NC mice (Fig 6A). SFZYD administration also significantly attenuated obesity-induced decreases in *AdipoQ* expression in AT (Fig 6A).

However, glucose uptake and insulin signaling related gene expression levels were not affected by SFYZD treatment. The mRNA expression levels of glucose transporter type 4 (*Slc2a4*) and insulin receptor substrate 1 (*Irs1*) were not changed by SFZYD treatment in AT, the major organ response for metabolizing glucose metabolism (Fig 6B). In animals, treatment with SFZYD for 4 weeks after the onset of obesity had no significant effects on fasting blood glucose levels, insulin levels, or homeostatic model assessment-insulin resistance (HOMA-IR) in obese mice (Figure A-C in S2 Fig). In addition, SFZYD treatment did not attenuate glucose intolerance in obese mice during the glucose tolerance test (GTT) (Figure D, E in S2 Fig).

### Alterations in metabolite levels are correlated with biochemical marker expression levels after SFZYD treatment

**Fig 7:**
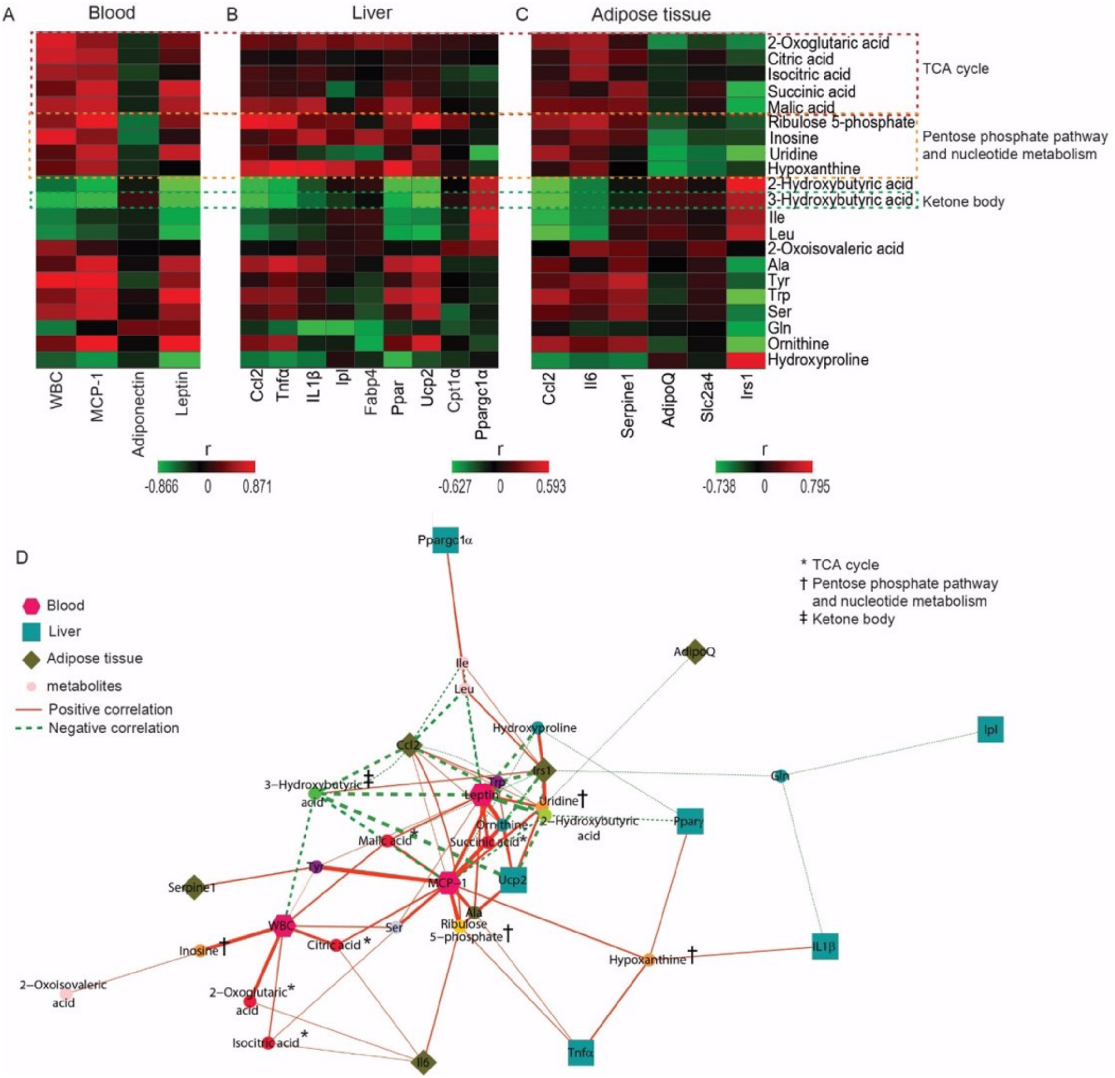
Altered metabolite levels are correlated with changes in biochemical marker levels. (A) Correlation heatmap showing the correlations between serum metabolite levels and blood parameter levels. (B) The mRNA expression levels of hepatic genes. (C) The mRNA expression levels of AT genes. (D) Correlation network comprising significant correlations (*p* value < 0.05) that was constructed using the edge-weighted spring-embedded layout algorithm in Cytoscape 3.2.1. The edges were weighted according to -|log *p*| values. The line and dashed line were indicative of positive and negative correlations, espectively, and the line-width represented |r| values in the correlation analysis (hexagon: blood parameter, square: liver gene, diamond: adipose gene, circle: serum metabolites).

Correlation analysis showed that the levels of serum metabolites associated with the TCA cycle, the pentose phosphate pathway and nucleotide metabolism (purine and pyrimidine metabolism) were positively correlated with WBC counts; serum MCP-1 levels; and hepatic *Ccl2*, *Tnfα*, *Il1β*, *Pparγ* and *Ucp2* levels, as well as AT *Ccl2*, *Il6* and *Serpine1* expression levels (Fig 7A–7D). However, the expression levels of 3-hydroxybutyric acid, a ketone body produced mainly in the liver, was negatively correlated with WBC counts; serum MCP-1 levels; and hepatic *Ccl2*, *Tnfα*, *Il1β*, *Pparγ*, and *Ucp2* expression levels, as well as AT *Ccl2*, *Il6* and *Serpine1* expression levels (Fig 7A–7D).

## Discussion

In this study, we first observed that administration of SFZYD, a remedy for treating BSS, significantly ameliorated systemic inflammation and hepatic steatosis. These effects were accompanied by serum metabolic pathway regulation. In addition to eliciting positive changes in phenotypes and metabolite patterns, SFZYD treatment regulates the TCA cycle, the pentose phosphate pathway and aromatic amino acid metabolism, as well as inflammatory cytokines and adipokines, effects that are related to improvements in systemic inflammation and hepatic steatosis.

In TKM and TCM, hepatic steatosis is attributed to blood stasis and Qi stagnation in the liver [6, 16]. Hepatic steatosis has been shown to be closely associated with chronic inflammation [9, 17]. The usefulness of inflammatory markers as potential diagnostic tools in non-alcoholic fatty liver disease (NAFLD) has also been investigated. Fatty liver disease is associated with increased TNF-*α* levels and decreased adiponectin levels, phenomena that correlate with inflammation and fibrosis severity [8, 9, 10, 11]. SFZYD administration restored serum adiponectin levels and increased hepatic *Tnfα* gene expression in obese mice to a normal level. Moreover, SFZYD treatment significantly decreased the expression levels of several inflammatory genes, including *Ccl2*, *Tnfα*, and *Il1β*, in the liver. SFZYD treatment also reduced the level of the circulating cytokine MCP-1, a product of *Ccl2* in serum, and the counts of circulating WBC without changing body weight, thus reducing systemic inflammation. Inflammatory gene expression in AT was significantly attenuated by SFZYD treatment. Shulan *et al*. recently reported that SFZYD improves inflammation-related metabolic disturbances in cold coagulation BSS by modifying phospholipids [18]. SFZYD has also been shown to effectively treat dysmenorrhea, a primary symptom of blood stasis, by regulating the expression of inflammatory cytokines, such as IL-1β, TNF-α, IL-10, IL-2 and IL-12, in a rat model [19]. Another decoction used to treat blood stasis and hypercoagulation in traditional medicine, Tongqiaohuoxue also ameliorates obesity-induced inflammation and prothrombotic states [5]. Emerging evidence indicates that BSS remedies have potential anti-inflammatory properties and primary effects in blood stasis.

Obesity-mediated hepatic steatosis was attenuated by reductions in hepatic lipid accumulation and TG levels in SFZYD-treated HFD mice; however no changes in organ weight were noted in these mice. TG accumulation in hepatocytes is a hallmark of hepatic steatosis resulting from imbalances between lipid uptake and exportation, as well as inflammation induced by circulating adipokines and cytokines [20]. In this study, histological observation and TG analysis of liver tissue specimens indicated that SFZYD administration ameliorated hepatic steatosis by reducing lipid accumulation. This finding was supported by our observation of decreases in serum TG levels after SFZYD treatment. Consistent with this finding, we found that SFZYD treatment induced similar significant decreases in the mRNA expression levels of *PPARγ* and its target genes, *Lpl* and *Fabp4*. Hepatic PPARγ is the main regulator of lipogenesis, fatty acid metabolism, and glucose metabolism and promotes lipid storage [21, 22]; thus, SFZYD-mediated regulation of the *PPARγ* gene may underlie the effects of SFZYD in liver steatosis. These results suggested that SFZYD administration improves hepatic steatosis by decreasing lipid metabolism-related marker expression levels and regulating inflammatory cytokine levels in obese mice.

NAFLD and type 2 diabetes mellitus frequently coexist in humans because they share the risk factors of excess adiposity and insulin resistance [23, 24]. In this study, we also measured insulin-resistance phenotype levels, *e*.*g*., fasting blood glucose levels, fasting insulin levels, HOMA-IR, and GTT. Treatment with SFZYD for 4 wks after the onset of obesity had no significant effects on fasting blood glucose levels, insulin levels, or HOMA-IR in obese mice (Figure A-C in S2 Fig). In addition, SFZYD treatment did not attenuate glucose intolerance in obese mice in the GTT, although it had a slight effect on glucose intolerance during the test. These results were consistent with those pertaining to the expression levels of the genes involved in glucose uptake and insulin signaling (Fig 6B). *Slc2a4* and *Irs1* expression levels were not affected by SFZYD treatment in AT, a major metabolic organ (Fig 6B). These results indicated that SFZYD treatment ameliorates obesity-mediated hepatic steatosis but not insulin resistance. However, an important issue worth discussing further is the mechanism underlying the effects of SFZYD in insulin resistance, as SFZYD was administered for only a short time in a chronic disease.

Circulating blood reflects systemic physiology; thus, we performed high-throughput metabolomics analyses of serum to comprehensively investigate the effects of SFZYD treatment on inflammation, as well as to better understand the biochemical mechanisms underlying the effects of SFZYD treatment on inflammation. HFD-fed mice had higher levels of TCA cycle intermediates, such as 2-oxoglutaric acid, citric acid, isocitric acid, malic acid and succinate, than NC mice, whereas SFZYD-treated mice had significantly lower levels of TCA cycle intermediates than HFD mice. In addition, the expression of the ketone body 3-hydroxybutyric acid was significantly lower in HFD mice than in NC mice but was significantly restored to its normal levels in SFZYD-treated HFD mice. All of these changes showed that SFZYD could correct HFD-induced metabolic disturbances, including HFD-induced TCA cycle alterations and decreases in β-oxidation energy production [25, 26]. In addition to eliciting the above changes in metabolite levels, ZYD also restored the expression of several hepatic genes, including *Ucp2*, *Cpt1α*, and *Ppargc1α*, which are related to mitochondrial dysfunction in obese mice. During hepatic steatosis progression, β-oxidation is disrupted, and mitochondrial oxidative capacity is lost, resulting decreased β-oxidation flux in the liver [27]. Thus, SFZYD administration reversed obesity-induced metabolic disturbances by balancing TCA cycle activity levels and β-oxidation energy production, phenomena accompanied by changes in the expression of related genes.

In general, the pentose phosphate pathway utilizes glucose to generate NADPH, which can be used in biosynthetic pathways, ROS production, and ribulose sugars for nucleotide biosynthesis [28]. In this study, the level of ribulose-5-phosphate, a pentose phosphate pathway product and intermediate, was significantly increased in the serum of obese mice. In addition, the levels of inosine, uridine, and hypoxanthine, which are intermediates of the *de novo* and salvage pathways of purine and pyrimidine synthesis, were increased in HFD mice, changes indicative of pentose phosphate pathway activation in obese mice. These findings were also consistent with those indicating that obese mice display greater phosphoribosyl pyrophosphate (PRPP) bioavailability and increased nucleotide requirements associated with subsequent increases in total RNA, DNA and adenine nucleotide production [29]. In particular, macrophages require NADPH, which is produced mainly by the pentose phosphate pathway [30, 31]. In obese mice, macrophages accumulate in expanded ATs and are major participants in AT inflammation and systemic insulin resistance [20]. We also observed crown-like structures (CLS) and immune cell infiltration and accumulation in the AT of obese mice (S3 Fig), changes that were reversed by SFZYD treatment. In addition, the abovementioned HFD-induced increases in serum MCP-1 and hepatic and AT *Ccl2* mRNA expression levels were attenuated by SFZYD. Ribulose 5-phosphate and hypoxanthine expression levels, which were correlated with the expression levels of the above markers, were also significantly altered by HFD and SFZYD treatment. Thus, SFZYD may ameliorate HFD-induced increases in pentose phosphate metabolite levels in obese mice through metabolic regulation.

SFZYD attenuated oxidative damage and inflammation by reducing intracellular ROS production and blocking H_2_O_2_ formation [32, 33]. Energy metabolism is altered in response to inflammation. Mills and O’Neill suggested that TCA cycle intermediates may represent a novel class of inflammatory regulators that act as key signals in multiple processes [34]. We observed that the levels of TCA intermediates were significantly correlated with those of the following inflammatory markers: WBC and MCP-1 in serum; *Ccl2*, *Tnfα*, *Il1β*, *PPARγ*, and *Ucp2* in the liver; and *Ccl2*, *Il6*, and *Serpine1* in AT (Fig 6A). Moreover, we observed that SFZYD decreased the level of liver TGs and altered the concentration of 2-hydroxybutyrate, a more sensitive index of liver function in obese patients and a surrogate biomarker for NAFLD in obese mice [35]. These results suggested that SFZYD may regulate systemic inflammation by regulating metabolites related to energy metabolism and inflammatory markers in the blood, liver, and AT.

Additionally, we also observed that Trp and Tyr levels were significantly higher in HFD mice than in NC mice, a phenomenon that was abolished by SFZYD administration. Serum Tyr concentrations are significantly increased in patients with liver injury [36]. Zhao *et al*. reported that urinary Trp levels are altered by treatment with an herbal preparation in a rat acute blood stasis model and suggested that Trp is a potential biomarker for BSS [37]. Based on these results, we concluded that SFZYD treatment attenuated HFD-induced disturbances in Trp and Tyr metabolism in obese mice and surmised that SFZYD prevents the metabolic changes associated with BSS and hepatic steatosis.

Studies have investigated the effects of multi-component drugs/herbal medicines using metabolomics strategies, and recent advances in metabolomics have enabled researchers to determine the effects of herbal remedies on the pathogenesis of various diseases through conjugated biomarker detection and metabolic pathway analysis. A recent study of a decoction of sini, an herbal supplement, detected and identified nineteen potential biomarkers in urine that may be used to assess the effects of the above decoction on myocardial infarction. These included succinate, citrate, 2-oxoglutarate (related to the TCA cycle) and L-tryptophan (related to tryptophan metabolism), whose levels were determined in myocardial infarction rats [38]. Na *et al*. studied the therapeutic effects of shenfu decoction in urine samples from rats with chronic heart failure and detected 16 TCA cycle metabolites whose levels are altered by treatment with the above decoction. These included citrate and L-tyrosine, and L-tryptophan, the latter two of which are related to tyrosine and tryptophan metabolism [39]. In this study of SFZYD decoction, we identified 27 metabolites whose expression level changes correlated with changes in biomarker networks after SFZYD treatment in systemic inflammation and hepatic steatosis. Taken together, these data suggested that SFZYD treatment regulates the TCA cycle, the pentose phosphate pathway and nucleotide metabolism pathways, and the findings of this study have provided us with insights into the effects of SFZYD treatment on systemic inflammation and hepatic steatosis.

In conclusion, the results of this study suggested that SFZYD, a BSS treatment, may ameliorate HFD-induced systemic inflammation and hepatic steatosis by regulating the expression of related metabolites and biomarkers. SFZYD treatment regulates the TCA cycle, the pentose phosphate pathway and aromatic amino acid metabolism, as well as inflammatory cytokines and adipokines, changes that are related to improvements in systemic inflammation and hepatic steatosis. This study showed that metabolomics approaches may be useful for exploring the effects of TKM therapy on various diseases and elucidating the therapeutic mechanisms through which herbal remedies exert their effects.

## Supporting information

S1 Fig PCA scores and loading plots for metabolic profiling in serum. (A) PCA scores and (B) loading plots of the serum metabolite concentrations obtained by targeted profiling of serum samples from mice on an NC diet (dot), an HFD (squares) and SFZYD with an HFD (triangles). The upper-left and lower-right sides of the loading plot are representative of higher metabolite levels in NC and HFD mice, respectively. The loading plot was produced from the scores plot, which shows differences in metabolite levels among the three groups (R2X = 0.598, Q2 = 0.158).
(PDF)

S2 Fig SFZYD treatment has no effects on insulin resistance. (A) Fasting blood glucose levels (B) Fasting blood insulin levels (C) HOMA-IR (D, E) glucose tolerance test (GTT) was measured during the 12th week of the HFD. Data are expressed as the mean ± SEM of 6–8 mice per group. *, *p* < 0.05; **, *p* < 0.01; ***, *p* < 0.001.
(PDF)

S3 Fig SFZYD treatment reduces immune cell accumulation in AT. Representative H&E-stained adipose tissue samples from mice on an NC diet, an HFD, and SFZYD with an HFD.
(PDF)
