# Supplementary figures and images for: Shaofu Zhuyu decoction ameliorates obesity-mediated hepatic steatosis and systemic inflammation by regulating metabolic pathways
Source: PLoS One. 2017 Jun 1;12(6):e0178514. doi: 10.1371/journal.pone.0178514 (PMC5453538; doi:10.1371/journal.pone.0178514)

**S1 fig.**

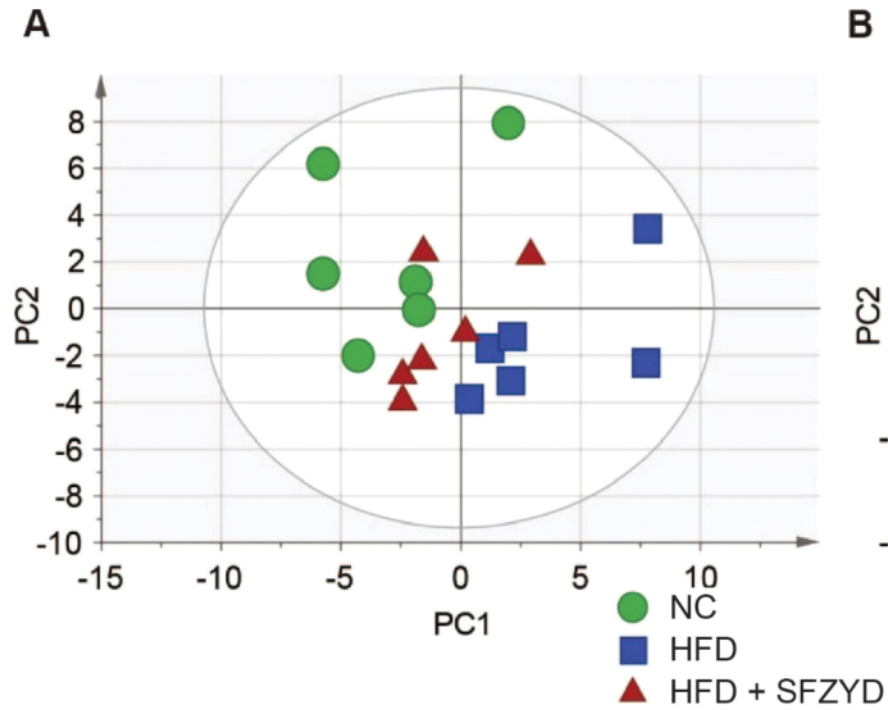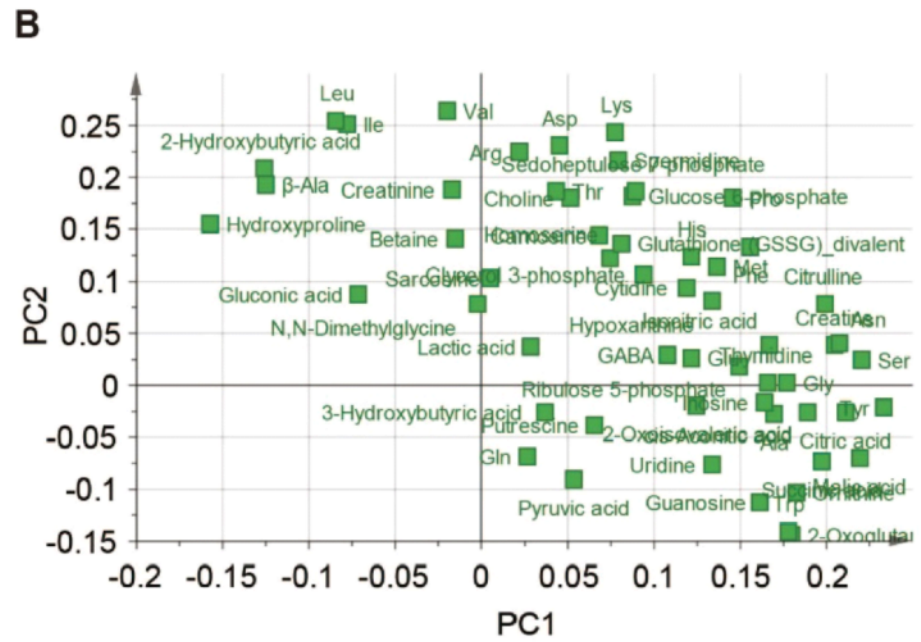

Supplement: S1 Fig — (A) PCA scores and (B) loading plots of the serum metabolite concentrations obtained by targeted profiling of serum samples from mice on an NC diet (dot), an HFD (squares) and SFZYD with an HFD (triangles). The upper-left and lower-right sides of the loading plot are representative of higher metabolite levels in NC and HFD mice, respectively. The loading plot was produced from the scores plot, which shows differences in metabolite levels among the three groups (R2X = 0.598, Q2 = 0.158). (PDF) [file pone.0178514.s001.pdf]

**S2 fig.**

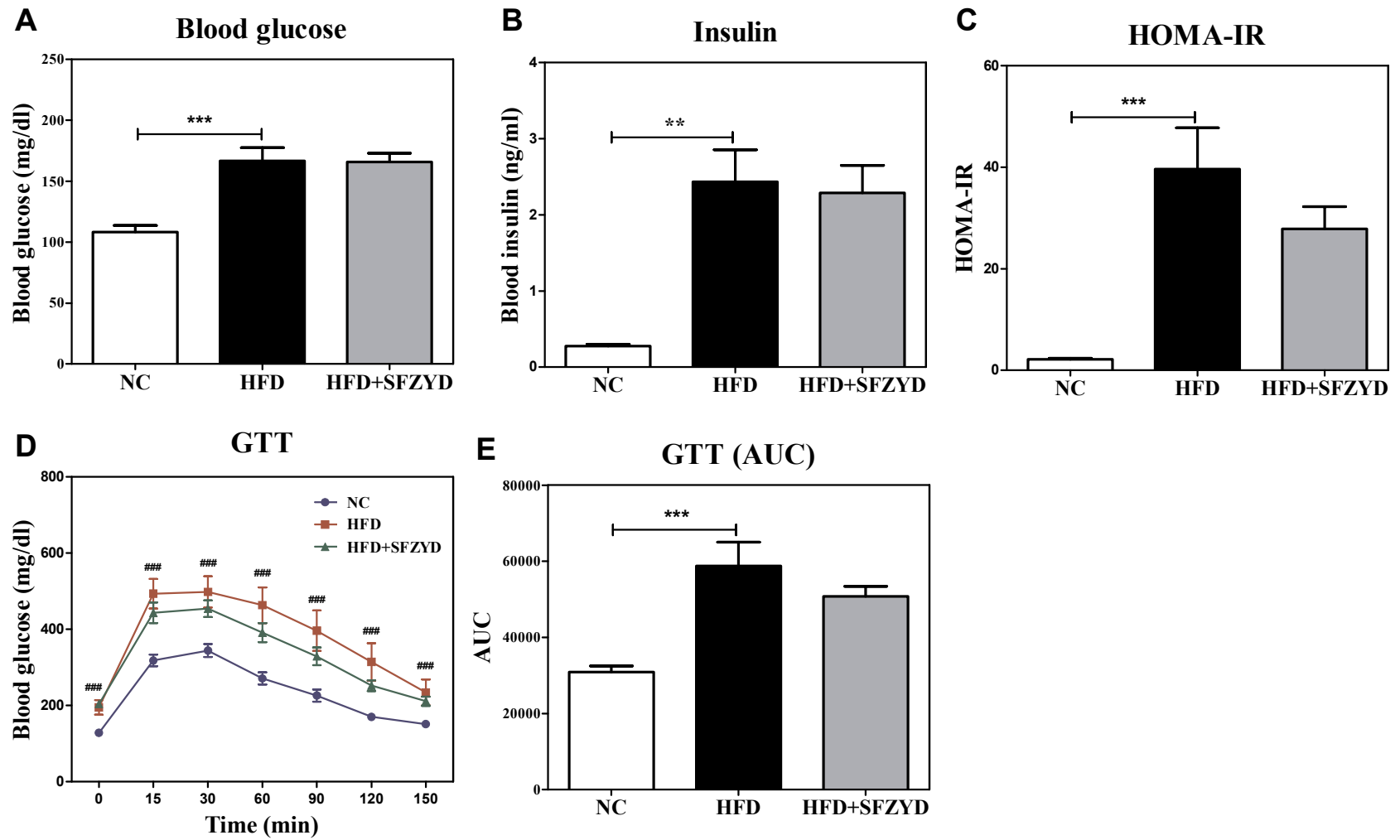

Supplement: S2 Fig — (A) Fasting blood glucose levels (B) Fasting blood insulin levels (C) HOMA-IR (D, E) glucose tolerance test (GTT) was measured during the 12th week of the HFD. Data are expressed as the mean ± SEM of 6–8 mice per group. *, p < 0.05; **, p < 0.01; ***, p < 0.001. (PDF) [file pone.0178514.s002.pdf]

**S3 fig.**

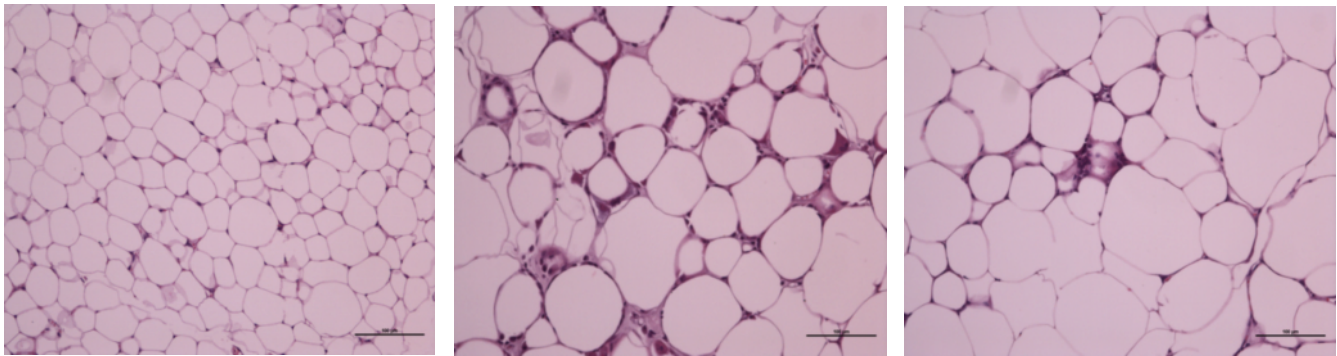

**NC**

**HFD**

**HFD + SFZYD**

Supplement: S3 Fig — Representative H&E-stained adipose tissue samples from mice on an NC diet, an HFD, and SFZYD with an HFD. (PDF) [file pone.0178514.s003.pdf]
